# Improved chromosome-level genome assembly of Indian sandalwood (*Santalum album*)

**DOI:** 10.1038/s41597-023-02849-x

**Published:** 2023-12-21

**Authors:** Xinhua Zhang, MingZhi Li, Zhan Bian, Xiaohong Chen, Yuan Li, Yuping Xiong, Lin Fang, Kunlin Wu, Songjun Zeng, Shuguang Jian, Rujiang Wang, Hai Ren, Jaime A. Teixeira da Silva, Guohua Ma

**Affiliations:** 1 Key Laboratory of South China Agricultural Plant Molecular Analysis and Genetic Improvement & Guangdong Provincial Key Laboratory of Applied Botany, South China Botanical Garden, Chinese Academy of Sciences, Guangzhou, 510650 China; 2 Bio&Data Biotechnologies Co. Ltd., Guangzhou, 510700 China; 3 Key Laboratory of Vegetation Restoration and Management of Degraded Ecosystems, South China Botanical Garden, Chinese Academy of Sciences, Guangzhou, 510650 China; 4 Key Laboratory of Plant Resources Conservation and Sustainable Utilization, South China Botanical Garden, Chinese Academy of Sciences, Guangzhou, 510650 China; 5 Independent researcher, Ikenobe 3011-2, Kagawa-Ken, 761-0799 Japan

**Keywords:** Plant biotechnology, Genomics

## Abstract

*Santalum album* is a well-known aromatic and medicinal plant that is highly valued for the essential oil (EO) extracted from its heartwood. In this study, we present a high-quality chromosome-level genome assembly of *S. album* after integrating PacBio Sequel, Illumina HiSeq paired-end and high-throughput chromosome conformation capture sequencing technologies. The assembled genome size is 207.39 M with a contig N50 of 7.33 M and scaffold N50 size of 18.31 M. Compared with three previously published sandalwood genomes, the N50 length of the genome assembly was longer. In total, 94.26% of the assembly was assigned to 10 pseudo-chromosomes, and the anchor rate far exceeded that of a recently released value. BUSCO analysis yielded a completeness score of 94.91%. In addition, we predicted 23,283 protein-coding genes, 89.68% of which were functionally annotated. This high-quality genome will provide a foundation for sandalwood functional genomics studies, and also for elucidating the genetic basis of EO biosynthesis in *S. album*.

## Background & Summary

The *Santalum* genus (family: Santalaceae; order: Santalales), broadly known as sandalwood, contains 15 species that are typically slow-growing hemiparasitic trees whose distribution ranges widely throughout India, Australia and the Pacific Islands^1^. Several *Santalum* species contain highly-prized sesquiterpene-rich essential oil (EO) in their aromatic heartwood, including *Santalum album* (Indian sandalwood), *S. yasi*, *S. spicatum*, *S. austrocaledonicum* and *S. insulare*^2^. The economic importance of Indian sandalwood is derived from its EO, which contains a high content of α- and β-santalol, and these account for as much as 6–7% of the EO on a fresh weight basis^3^. Even though the broad genetic center of origin lies in tropical and temperate regions of India, Indian sandalwood (and to a lesser extent, other sandalwood species) is now extensively cultivated in South China, Sri Lanka, Indonesia, Malaysia, the Philippines and Northern Australia^4^. Indian sandalwood has held an important societal status in India since 1772 due to its aromatically fragrant EO^5^. The EO also has a long (~4,000 years) and acclaimed history in perfumery, medicine, religion, and other cultural applications^6^. The EO, or compounds within it, have displayed antioxidant, antitumour, antidepressive and anti-inflammatory activity^7,8^. Excessive demand of Indian sandalwood due to its commercial value resulted in the unsustainable exploitation of natural stands, and absent the establishment of new plantations, this led to a rapid increase in market prices^9^. Heartwood-derived EO was priced at more than $5,000/kg on the international market^10^.

As a result of the cultural and commercial importance of heartwood-derived EO, scientists have been eager to research its phytochemical characteristics. To date, most work has focused on *S. album* with the highest quality EO. Previous research identified over 100 terpenoids in *S. album* EO, about 80% of which consisted of (*Z*)-α-santalol and (*Z*)-β-santalol^11^. For at least a decade, attention has been paid to understanding the mechanisms associated with the biosynthetic pathway of the EO in *S. album*. Some key enzymes, including santalene/bergamotene synthase, SaCYP736A167 and SaCYP76Fs 37–43, have been characterized in *S. album*^12–14^. Others such as acetyl-CoA acetyltransferase, hydroxymethylglutaryl-CoA synthase, mevalonate kinase and phosphomevalonate kinase in the mevalonate pathway were also reported^15,16^. Two *S. album* genomes were generated based on Illumina short-read sequencing^17,18^. Recently, chromosome-level genome assemblies of *S. album* and *S. yasi* were documented^19^. However, knowledge of the genetic basis of EO biosynthesis in sandalwood trees is scarce.

**Fig. 1:**
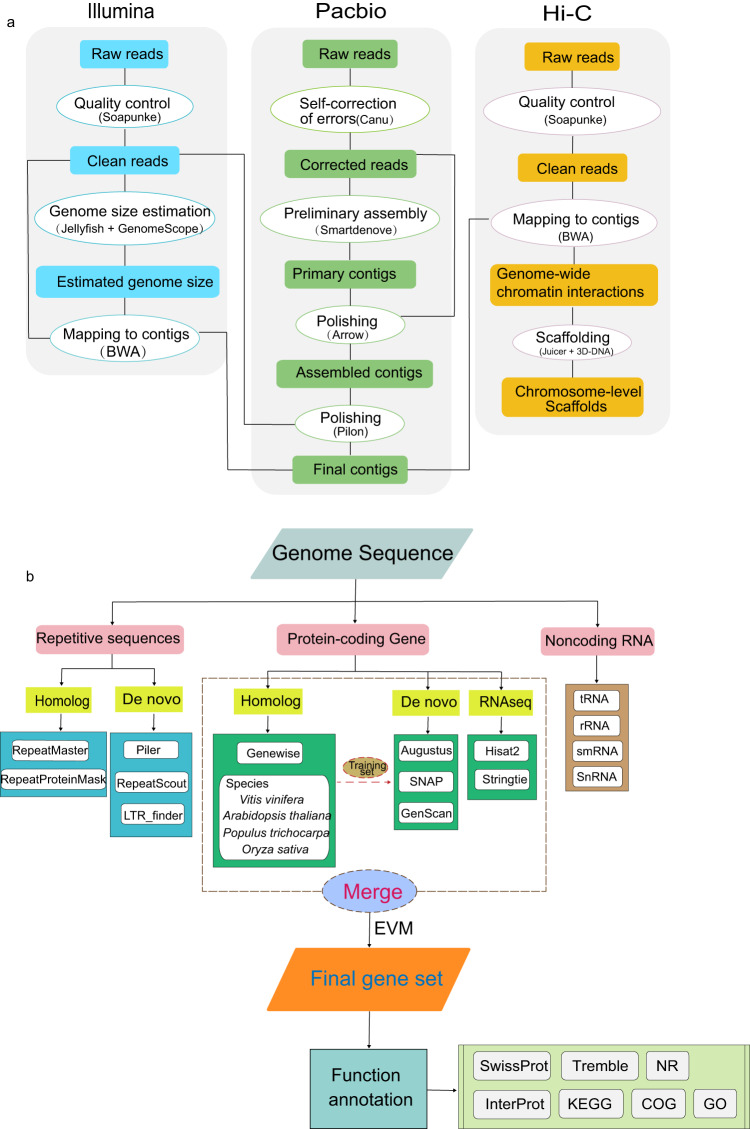
Flowchart of sequencing and assembly for the *Santalum album* genome.

**Fig. 2:**
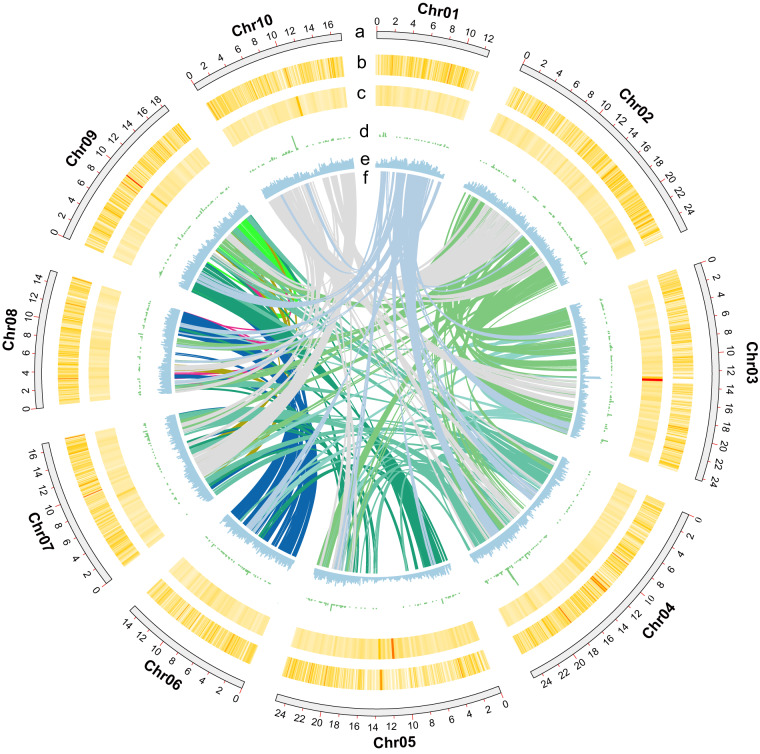
Genomic features of *S. album*. Circos plot from the outer to the inner layers represents the following: (**a**) Genomic landscape of the 10 assembled pseudo-chromosomes (Mb); (**b**) the GC density; (**c**) non-coding RNA; (**d**) transposable elements (TEs); (**e**) distribution of the density of genes and (**f**) syntenic blocks.

**Fig. 3:**
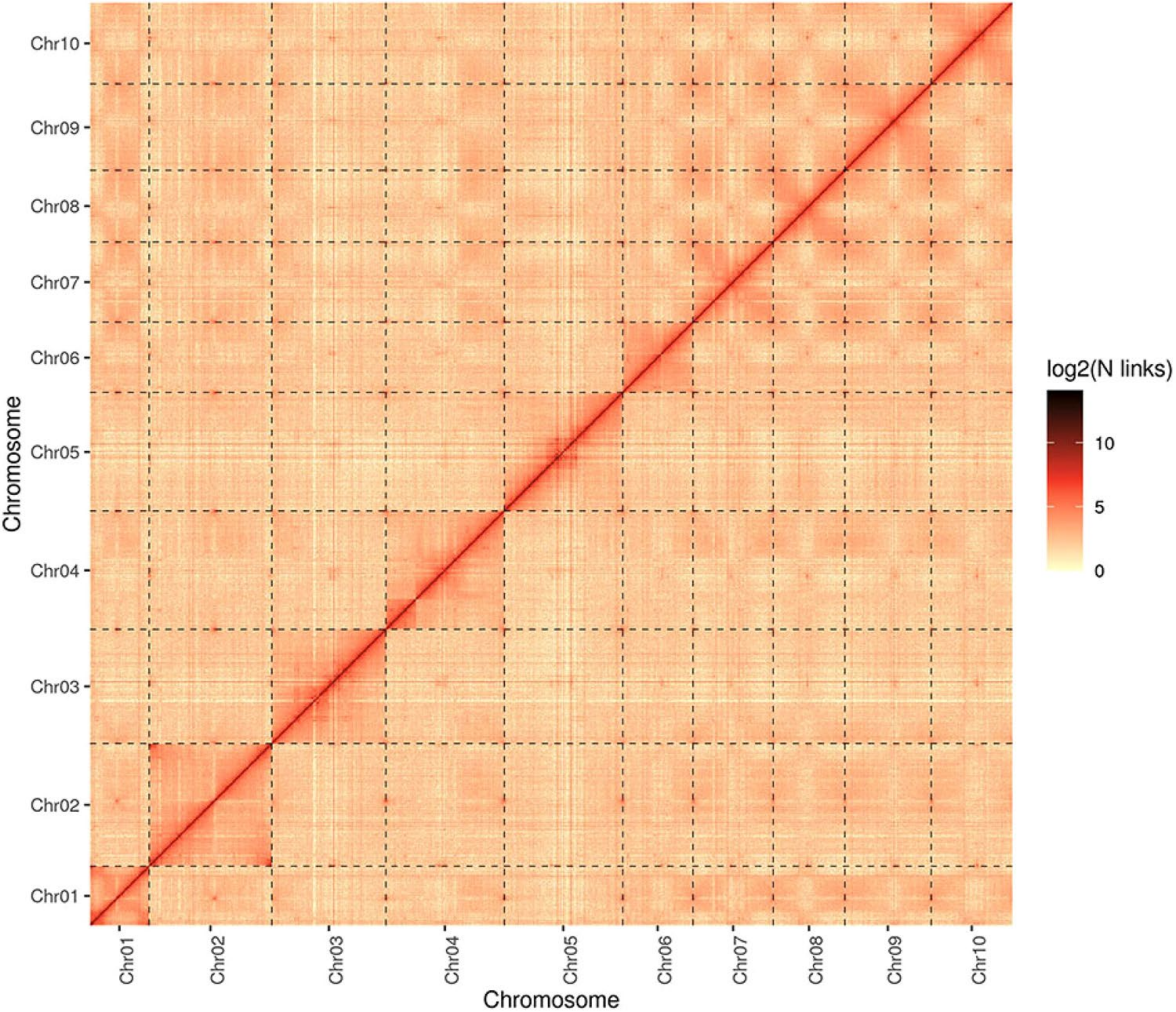
Heat map of genome-wide Hi-C intra-chromosome interactions in *S. album*. The image represents validation of the Hi-C-assisted pseudo-chromosome assembly by calculation of the thermal interaction correlation.

A total of approximately 144.28 Gb of clean reads comprising 40.99 Gb of PacBio long reads, 37.84 Gb of Illumina reads and 65.45 Gb of Hi-C reads, were generated in this study (Table 1). A *K-*mer distribution analysis (*K* = 17) revealed that the estimated size of the *S. album* genome is 246.55 Mb, with a heterozygosity rate of 0.56% (Fig. S1; Table S1). A *de novo* assembly strategy combining PacBio long reads and Illumina paired-end reads resulted in an assembly of 207.39 Mb, with 7.33 Mb of N50 contigs (Fig. 1; Table 2; Table S2). High-quality Hi-C data were then used to further super-scaffold the genome assembly. Finally, a reference genome of *S. album* at the chromosome level was obtained by anchoring contigs 195.49 Mb in size into 10 chromosomes with lengths ranging between 12.54 Mb (Chr01) and 25.96 Mb (Chr02) (Figs. 2, 3; Table 3; Tables S3, S4). In terms of total length, the chromosomes accounted for 94.26% of the genome sequence, with a N50 scaffold of 18.31 Mb (Table 4), while the anchor rate was higher than that documented by Hong *et al*.^19^, with a mounting rate of 90.78% (Table 5). Mapped reads based on Illumina sequencing data to the assembled contigs accounted for as much as 96.83% of the total (Table S5). We assessed core gene statistics using Benchmarking Universal Single-Copy Orthologues (BUSCO)^20^ to verify the sensitivity of gene prediction and completeness. The result indicates that 94.91% of plant sets (1300 out of 1375 BUSCOs) were identified as complete (Table 6). The GC content was 37% (Table 2; Fig. S2). Collectively, these statistics and findings of the genome’s quality confirm that this chromosome-level genome assembly is complete and of high quality.

**Table 1:** Statistics of sequencing data for the *S. album* genome.

Libraries	Raw data (Gb)	Clean base (Gb)	Insert size	Read length (bp)	Sequence coverage (X)	Maximum length (bp)	N50 length (bp)
Illumina Hiseq XTen	40.13	37.84	350 bp	150	145.52		
PacBio	40.99		20 Kb	13985.57	164	238,867	21,444
Hi-C	68.08	65.45	350 bp	150			
RNA-seq	12.18	10.99		100			
Total	160.19

**Table 2:** Statistics of assembly and annotation of *S. album* genome.

Characteristics	Statistics
Estimated genome size (by k-mer analysis) (Mb)	246.55
Assembled genome size (Mb)	207.39
Length loaded on pseudochromosomes (Mb)	195.49
No. of pseudochromosomes	10
Longest scaffold (bp)	25,964,583
N50 of contigs (bp)	7,325,660
N50 of scaffolds (bp)	18,311,000
Anchored rate (%)	94.26
GC content (%)	37
Complete BUSCOs (%)	94.91
Repeat region % of assembly	27.55
redicted gene models	23,283
Average coding sequence length (bp)	1189
Average No. of exons per gene	5.4
Average exon length (bp)	220.36
No. of functions annotated	20,880

**Table 3:** Chromosome lengths of the assembled sandalwood genome.

Pseudochromosome name	Chromosome length (bp)
SaChr1	12,536,236
SaChr2	25,964,583
SaChr3	24,276,000
SaChr4	25,041,609
SaChr5	25,102,000
SaChr6	14,974,638
SaChr7	16,909,640
SaChr8	15,281,464
SaChr9	18,311,000
SaChr10	17,148,921
Unanchored	11,902,219

**Table 4:** Assembly improvement using Hi-C.

	Contig		Scaffold	
	Length (bp)	Number	Length (bp)	Number
N50	7,325,660	12	18,311,000	5
N90	1,378,072	37	12,536,236	10
Total	207,394,310	/	207,778,310	/
Anchored size	195,492,091	/		
Anchored rate (%)		94.26

**Table 5:** Comparisons of genome assemblies and annotations.

	In this study	Mahesh *et al*.^17^	Dasgupta *et al*.^18^	Hong *et al*.^19^
Genome size (Mb)	207.39	220.961	286	229.59
N50 (Mb)	7.33	0.46	0.012	5.93
GC content (%)	37	34.38	—	36
Complete BUSCOs (%)	94.91	94.38	—	92.50
Repeat region (%)	27.55	27.42	12.52	28.93
Predicted gene models	23,283	38,119	37,500	21,673
No. of functions annotated	20,880	18,533	25,000	20,635
Anchored rate (%)	94.26	—	—	90.78

**Table 6:** BUSCO assessment of *S. album* genome assembly and annotation.

Type	Assembly				Annotation	
	PacBio		Hi-C			
	Count	Percentage (%)	Count	Percentage (%)	Count	Percentage (%)
Complete BUSCOs	1305	94.91	1300	94.55	1251	90.98
Complete and single-copy BUSCOs	1261	91.71	1264	91.93	1206	87.71
Complete and duplicated BUSCOs	44	3.20	36	2.62	45	3.27
Fragmented BUSCOs	16	1.16	18	1.31	49	3.56
Missing BUSCOs	54	3.93	57	4.15	75	5.45
Total BUSCO groups searched	1375	100	1375	100	1375	100

**Fig. 4:**
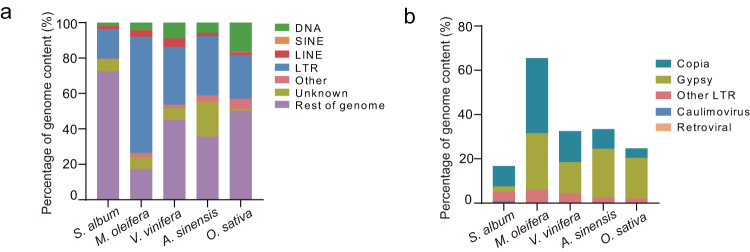
Transposable elements in *S. album*. (**a**) Proportions of TEs among genomes of *S. album*, *Malania oleifera*, *Vitis vinifera*, *Aquilaria sinensis* and *Oryza sativa*. (**b**) Percentage of genome content comprising LTR elements for these five plant species.

Transposable elements (TEs) are the main mechanistic drivers of genome evolution^21^. The Indian sandalwood genome harbored a total of 57.15 Mb of TEs, representing approximately 27.55% of the assembly (Tables S6–S8). Compared with four plant species (*Malania oleifera*, *Vitis vinifera*, *Aquilaria sinensis* and *Oryza sativa*) whose genomes were sequenced, sandalwood had the smallest genome and the lowest content of repetitive DNA (Fig. 4; Fig. S3; Table S9). Long terminal repeat (LTR) retrotransposons represented the greatest proportion of repeated content in these plants’ genomes, accounting for 16.75% of the *S. album* genome. Copia-LTR repeats dominate the sandalwood tree genome, contributing about 20.67 Mb (9.96%), and were 3.78-fold more abundant than Gypsy-LTR, accounting for 5.46 Mb (2.63%). In contrast, Gypsy-LTR was the most abundant repeat class in *V. vinifera*, *A. sinensis* and *O. sativa* and a nearly equal amount of Copia (29.51%) and Gypsy (28.15%) LTR-RTs were annotated in the *M. oleifera* genome.

After masking repeat sequences, we predicted the presence of 23,283 protein-coding genes by integrating *de novo* predictions, homology-based predictions, and transcriptomic data, with an average length of 3,812 bp, an average coding sequence length of 1,188 bp, and an average of 5.4 exons per gene in *S. album* (Fig. S4 and Tables S10, S11). About 89.68% of protein-coding genes had significant hits in several functional annotation databases (SwissProt, TrEMBL, InterPro, KEGG, Nr, COG and GO) (Fig. S5; Table S12). Among them, 1,368 genes encoding transcription factors (TFs) were predicted and classified into 58 gene families (Fig. S6). In addition, noncoding RNA (ncRNA) genes, including 65 microRNAs (miRNAs), 495 transfer RNA (tRNA), 585 ribosomal RNA (rRNA) and 257 small nuclear RNA (snRNA) genes, were identified in the genome (Table S13).

**Fig. 5:**
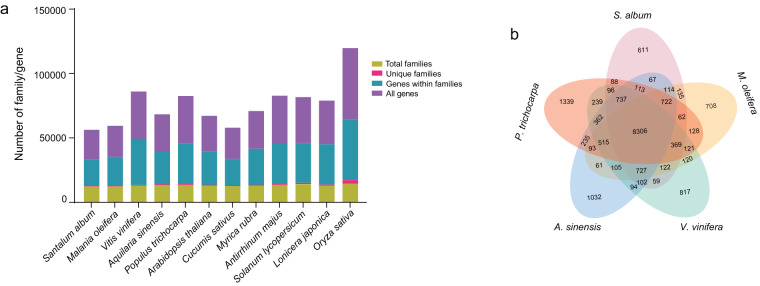
Comparative genomics analysis. (**a**) Statistics of gene families and all genes in *S. album* and other representative plant species. (**b**) Venn diagram shows the shared and unique gene families among Indian sandalwood and four other plant species.

We compared the *S. album* assembly with sequenced genomes from 11 other plants, including *M. oleifera*, *V. vinifera*, *Arabidopsis thaliana*, *Populus trichocarpa*, *A. sinensis*, *Cucumis sativus*, *Myrica rubra*, *Antirrhinum majus*, *Solanum lycopersicum*, *Lonicera japonica* and *O. sativa* (Table S14). Based on gene family clustering analysis, 23,283 genes in *S. album* clustered into 12,430 gene families; 12,067 gene families were shared among all 12 plant species, and 344 families were unique to *S. album* (Fig. 5).

In summary, this study presents a greatly improved sandalwood genome version, both in terms of completeness and accuracy, compared with two previously published *S. album* genomes that were obtained exclusively from short-reads^17,18^. In addition, not only is the contig N50 of our genome longer than that of a recently released genome^19^, the anchor rate of the genome assembly onto pseudo-chromosomes has also improved significantly. The chromosome-level *S. album* genome described in this paper provides a highly accurate and contiguous reference of genome sequences. Our study provides insight into the evolution of *S. album*, and presents a valuable genomic resource for further elucidating the genetic basis of EO biosynthesis in sandalwood trees.

## Methods

### Sample collection and DNA extraction

Fresh leaf tissues were collected from a 10-year-old tree grown at the South China Botanical Garden, CAS, Guangzhou, China in May, 2020 and immediately frozen in liquid nitrogen and stored at −80 °C. High-quality genomic DNA was extracted using a modified CTAB method^22^.

### Genome sequencing and assembly

A paired-end library with insert lengths of 350 bp was constructed using the Illumina TruSeq library construction kit according to the manufacturer’s instructions. The constructed library was sequenced on the Illumina HiSeq X Ten platform (Illumina, San Diego, CA, USA) at the Beijing Genomics Institute (BGI). For PacBio sequencing, one library with a 20 kb insert size was constructed with the SMRTbell Template Prep Kit 1.0 according to the PacBio standard protocol. In brief, high-quality DNA was fragmented and concentrated. The fragments were purified by beads, damage was repaired, and resulting fragments were used as the 20 kb SMRTbell templates. PacBio long reads were sequenced for two SMRT cells on the PacBio Sequel System (Pacific Biosciences, CA, USA) at the BGI.

Errors in the PacBio SMRT sequences were initially corrected by Canu (v1.8)^23^ with the following parameters: corOutCoverage = 50, useGrid = true. Corrected reads were then assembled to primary contigs using SMARTdenovo (https://github.com/ruanjue/smartdenovo) with the following parameters: -c 1 -t 15 -J 5000 -k 17. The draft genome was polished using Arrow software (https://github.com/PacificBiosciences/Genomic-Consensus) based on corrected PacBio long reads with options parameters. To increase the accuracy of the assembly, Illumina short reads were recruited to correct the assembled contigs through the Pilon program (https://github.com/broadinstitute/pilon). The quality of genome assembly was assessed using BUSCO^20^.

### Genome size estimation

The size of the *S. album* genome was estimated by using *k*-mer (k = 17) distribution analysis with Jellyfish^24^ using 350 bp Illumina pair-end reads. The Illumina reads were first trimmed to remove adaptors and reads with >10% ambiguous or >20% low-quality bases using the SOAPnuke package (v1.6.5)^25^ with parameters “-n 0.05 -l 15 -q 0.2 -Q 2 −5 1”. In this analysis, genome size = *k*_num_/*k*_depth_, where *k*_num_ is the total number of *k*-mers, and *k*_depth_ is the expected depth of *k*-mers. The size of the *S. album* genome was estimated as 246.55 Mb with the total number of 17-mers = ∼3.3 × 10^10^ and their main peak at a depth of 145.52, using GenomeScope^26^.

### Hi-C scaffolding

About 5 g of fresh young leaf tissue from living plants was used to construct the Hi-C sequencing library. Samples were crosslinked using 37% formaldehyde to yield a 2% final concentration, mixed gently and incubated at room temperature (RT) for 10 min on plates that were gently rotated every 2 min. Then, 2.5 mL of 2.5 M glycine was added to quench the crosslinks, mixed well, incubated at RT for 5 min, then incubated on ice for 15 min to stop crosslinking completely. Cross-linked DNA was digested with *Mbo*I endonuclease overnight. The sticky ends of the digested fragments were biotinylated, diluted, and randomly ligated to each other to form chimeric junctions. Following ligation, a protease was used to remove the crosslinks and DNA was purified using the Qiagen MinElute PCR Purification Kit according to the manufacturer’s protocols. Finally, the Hi-C library with an insert size of 350 bp was constructed and sequenced on the BGISEQ-500 sequencer (BGI, Beijing China) with 2 × 150-bp reads at the BGI.

Hi-C raw reads were filtered using SOAPnuke (v1.6.5)^25^ with the following parameters (-n 0.05 -l 15 -q 0.2 -G -Q 2 −5 0) to obtain clean reads. Clean reads were first aligned to the contig-level sandalwood genome using Bowtie2 (v2.2.5)^27^ with the following parameters: “GLOBAL_OPTIONS = --very-sensitive -L 30 --score-min L, −0.6, −0.2–end-to-end –reorder” and “LOCAL_OPTIONS = --very-sensitive -L 20 --score-min L, −0.6, −0.2 --end-to-end --reorder”. The ratio of mapped reads to total reads reached 91.14% and 91.16%, respectively (Supplemental Table 1). The Hi-C sequence data obtained from global mapped reads were further qualified with HiC-Pro (v2.5.0)^28^, in which the unique mapped read pairs were selected. Final valid interaction pairs were obtained after removing duplicated pairs. The Hi-C data were then aligned and the misassembled reads were deleted by the Juicer pipeline^29^ to obtain unique reads. 3D *de novo* assembly (3D-DNA) software was applied to cluster, order and orient the filtered contigs onto chromosomes^30^. The completeness of the genome assembly was evaluated using BUSCO^20^. In addition, the paired-end Illumina short reads were mapped to the genome assembly using BWA-MEM (v0.7.12)^31^ to assess the integrity and accuracy of the genome.

### Transcriptome sequencing

To facilitate gene model prediction, all tissues (leaves, flowers, fruits, heartwood and roots) were obtained from 10-year-old sandalwood trees for transcriptome sequencing. Leaves, flowers and fruits were harvested separately. Wood shavings of heartwood and root tissues were obtained using a Hagloff wood borer as described previously^32^. Total RNA was extracted followed an established method^33^. RNA (2 µg) from each sample was pooled to construct cDNA libraries, which were sequenced on BGISEQ-500 with PE100. Clean reads from all tissues were obtained by removing adaptor sequences and filtering low-quality reads with SOAPnuke^25^ with the following parameters: “-l 15 -q 0.5 -n 0.1”.

### Genome model prediction and functional annotations

Repeat sequences in the *S. album* genome were annotated by integrating *de novo* and homology-based approaches. First, a *de novo* library from the assembled genome was constructed using LTR_Finder (v1.0.6)^34^ (http://tlife.fudan.edu.cn/ltr_finder/), Piler^35^ (http://www.drive5.com/piler/), and RepeatScout^36^ (http://www.repeatmasker.org/). Then, this *de novo* repeat database together with the Repbase TE library (http://www.girinst.org/repbase) were used to identify repeats by RepeatMasker (v4.0.7)^37^ and to identify repeat related proteins by RepeatProteinMask (v4.0.7) (http://www.repeatmasker.org/). We also annotated the non-interspersed repeat sequences, including low complexity repeats, satellites and simple repeats, with RepeatMasker (v4.0.7).

Protein-encoding gene models were predicted using *ab initio*, homology-based and RNA-seq-based pipelines based on the repeat-masked genome. Firstly, the protein sequences from grape (*V. vinifera*), Arabidopsis (*A. thaliana*), poplar (*P. trichocarpa*) and rice (*O. sativa*) were downloaded from the Plant Genome Database (PlantGDB; http://www.plantgdb.org/), then aligned to the sandalwood genome assembly using Blast with the following parameters: “blastall -F F -m 8 –p tblastn -e 1e-05 -a 5”. Secondly, GeneWise (v2.4.1)^38^ was used to align them against corresponding proteins to determine gene structures with the following parameters: “genewise -trev -sum -genesf -gff”. For the *de novo* prediction, Augustus^39^ and SNAP^40^ were applied. Furthermore, Genscan^41^ was used for *de novo* predictions with gene model parameters trained from Arabidopsis. Clean RNA-seq reads were aligned to the sandalwood genome with HISAT2 (v2.0.4) with the parameters “--phred64 --sensitive --no-discordant–no-mixed -I 1 -X 1000 –dta”^42^ and transcripts were predicted by StringTie (v1.0.4)^43^ with parameters “-f 0.3 -j 3 -c 5 -g 100 -s 10000 -p 8”. Finally, gene models from *ab initio*, homology-based, and transcriptome-based predictions were merged by EVidenceModeler software (v1.1.1)^44^. To validate the quality of the gene predictions, we compared the length distribution of protein-coding genes, coding sequences, exons and introns between sandalwood, grape, Arabidopsis and poplar. Completeness of the final gene set was assessed with BUSCO^20^. These genes were named according to the nomenclature used for Arabidopsis (Arabidopsis Genome Initiative, 2000) to indicate the relative positions of genes on the pseudo-chromosomes.

The functions of protein-coding genes were identified by BLASTP (v2.2.31) searches against SwissProt (http://www.uniprot.org/), GO (http://geneontology.org/page/go-database), KEGG (http://www.genome.jp/kegg/) and NR (http://ftp.ncbi.nlm.nih.gov/) databases. The predicted proteome was also assigned to TF families based on PlantTFDB (v5.0) (http://planttfdb.gao-lab.org/). *S. album* noncoding RNAs were annotated by tRNAscan-SE^45^ for tRNA, BLASTN for rRNA, and INFERNAL for miRNA and snRNA^46^.

In addition, we used OrthoFinder (v2.3.1)^47^ to identify gene families from *S. album* and 11 representative plant genomes, 10 eudicots including *M. oleifera* in the Santalales, *V. vinifera*, *A. thaliana*, *P. trichocarpa*, *A. sinensis*, *C. sativus*, *M. rubra*, *A. majus*, *S. lycopersicum* and *L. japonica*, and one monocot, *O. sativa*.

## Data Records

The raw sequencing data have been deposited in the Genome Sequence Archive at the National Genomics Data Center (NGDC, https://ngdc.cncb.ac.cn/), Beijing Institute of Genomics, Chinese Academy of Sciences/China National Center for Bioinformation, under accession code CRA009778, including the PacBio reads^48^, Illumina short reads^49^, Hi-C Illumina reads^50^ and transcriptome reads^51^, which only these data were associated with this study. This Whole Genome Shotgun project has been deposited at DDBJ/ENA/GenBank under the accession JAXCHL000000000^52^. The version described in this paper is version JAXCHL010000000. The chromosome-level assembled genome sequences and annotation were deposited in the Figshare database^53^.

## Technical Validation

To evaluate the completeness of the sandalwood assembly, we first mapped Illumina short-reads to the PacBio long read-based assembly to obtain 100% coverage. Then, BUSCO was employed to assess the assembly’s completeness. A total of 1,305 complete BUSCOs (94.91%) out of the 1,375 BUSCO groups were identified, including 1,261 complete and single-copy BUSCOs and 44 complete and duplicated BUSCOs, suggesting a remarkably complete assembly of the *S. album* genome. Moreover, we anchored the Hi-C data to the 10 pseudo-chromosomes, and then analyzed and visualized the Hi-C data. The paired-end Illumina short reads were mapped to the genome assembly to yield a 96.83% mapping rate. The signal intensities of interaction between the two bins were clearly divided into 10 distinct groups (Fig. 3), indicating the high-quality nature of the pseudo-chromosomes’ assembly. Finally, a list of chromosome ID conversions between assembled pseudo-chromosomes documented in this study and in a previous report^19^, has been compiled in Table S15.

### Supplementary information

Supplementary Information

## Data Availability

All bioinformatic tools used in this study followed the corresponding manuals and protocols. The versions and code/parameters of software are described in the Methods. Default parameters were employed if no detailed parameters were mentioned for the software used in this study.
