# Supplementary material for: Improved chromosome-level genome assembly of Indian sandalwood (Santalum album)
Source: Sci Data. 2023 Dec 21;10:921. doi: 10.1038/s41597-023-02849-x (PMC10739715; doi:10.1038/s41597-023-02849-x)
Supplement: Supplementary file 1 — Supplementary Information [file 41597_2023_2849_MOESM1_ESM.pdf]

Supplemental Information

Improved chromosome-level genome assembly of Indian sandalwood (*Santalum album*)

Xinhua Zhang[1#*], MingZhi Li[2#], Zhan Bian[1], Xiaohong Chen[1], Yuan Li[1], Yuping Xiong[1], Lin Fang[1], Kunlin Wu[1], Songjun Zeng[1], Shuguang Jian[3], Rujiang Wang[4], Hai Ren[3], Jaime A. Teixeira da Silva[5], Guohua Ma[1*]

[1]Key Laboratory of South China Agricultural Plant Molecular Analysis and Genetic Improvement & Guangdong Provincial Key Laboratory of Applied Botany, South China Botanical Garden, Chinese Academy of Sciences, Guangzhou, 510650, China

[2]Bio&Data Biotechnologies Co. Ltd., Guangzhou, 510700, China

[3]Key Laboratory of Vegetation Restoration and Management of Degraded Ecosystems, South China Botanical Garden, Chinese Academy of Sciences, Guangzhou, 510650, China

[4]Key Laboratory of Plant Resources Conservation and Sustainable Utilization, South China Botanical Garden, Chinese Academy of Sciences, Guangzhou, 510650, China

[5]Independent researcher, Ikenobe 3011-2, Kagawa-Ken, 761-0799, Japan

[#]Xinhua Zhang and MingZhi Li contributed equally to this work

[*]Co-corresponding authors:

Xinhua Zhang, xhzhang@scib.ac.cn

Guohua Ma, magh@scib.ac.cn

Table of Contents:

Table S1	Page 3
Table S2	Page 4
Table S3	Page 5
Table S4	Page 6
Table S5	Page 7
Table S6	Page 8
Table S7	Page 9
Table S8	Page 10
Table S9	Page 11
Table S10	Page 12
Table S11	Page 13
Table S12	Page 14
Table S13	Page 15
Table S14	Page 16
Table S15	Page 17
Figure S1	Page 18
Figure S2	Page 19
Figure S3	Page 20
Figure S4	Page 21
Figure S5	Page 22
Figure S6	Page 23

Table S1. Statistics of the genomic characteristics of *S. album* obtained by genome survey analysis

K-mer	K-mer number	Genome size (Mb)	Heterozygous ratio (%)	Repeat (%)	Used bases	Used reads	K-mer depth
17	33,400,952,560	246.55268	0.56	1.48	37,389,126,000	249,260,840	145.5224

Table S2. Assembly of the *S. album* genome

	Canu + SMARTdenovo		Polish with PacBio data		Pilon with Illumula data	
	**Size (bp)**	**Number**	**Size (bp)**	**Number**	**Size (bp)**	**Number**
N90	1,380,911	37	1,380,845	37	1,378,072	37
N80	2,719,189	26	2,720,727	26	2,719,566	26
N70	4,135,395	20	4,138,185	20	4,134,551	20
N60	5,590,127	16	5,592,309	16	5,587,693	16
N50	7,326,248	12	7,329,047	12	7,325,660	12
Longest	11,246,021	---	11,254,383	---	11,247,869	---
Total size	207,466,477	---	207,573,393	---	207,394,310	---
≥ 100 bp	---	127	---	127	---	127
≥ 2 kb	---	127	---	127	---	127

Table S3. Statistics of clean reads mapped to the genome in Hi-C sequencing

**Type**	**R1**	**R2**
Total reads	218,299,377	218,299,377
Mapped reads	198,953,827	198,992,175
Global mapped reads	137,889,783	135,403,583
Local mapped reads	61,064,044	63,588,592
Mapping ratio (%)	91.14%	91.16%

Table S4. Assessment of the quality of the Hi-C construction library

**Analysis**	**Type**	**Ratio (%)**
Unique mapped read pairs	Unique pairs	41.10
	Multiple and singleton pairs	57.78
	Unmapped pairs	1.11
Valid interaction pairs	Valid interaction pairs	88.49
	Invalid pairs	11.51
Final valid interaction pairs	Valid interaction pairs	88.74
	Duplicates	11.26
Total*	218,299,377 * 41.10% * 88.49% * 88.74% = 70,458,079	

*Total = the ratio of reported unique pairs × the ratio of valid interaction pairs × (1 – the ratio of duplicated pairs)

Table S5. Assessment of genome integrity based on Illumina sequencing data

	**Number**	**Ratio (%)**
Total reads	258,087,186	100
Mapped reads	249,911,873	96.83
Mapped and paired reads	223,044,078	86.42

Table S6. Statistics of repeat sequences in the *S. album* genome

Type	Repeat size (bp)	% of genome
TRF	6,260,240	3.08
RepeatMasker	10,209,758	4.92
RepeatProteinMask	8,663,620	4.18
De novo	55,998,927	26.99
Total	60,451,593	29.14

Table S7. Classification of repetitive sequences in the *S. album* genome

Type	Repbase TEs		TE proteins		De novo		Combined TEs	
	**Length (bp)**	**% of genome**	**Length (bp)**	**% in genome**	**Length (bp)**	**% in genome**	**Length (bp)**	**% in genome**
DNA	2,028,670	0.977916	120,547	0.05811	8,525,332	4.109617	9,857,090	4.751588
LINE	929,467	0.448048	507,447	0.24461	3,022,300	1.456893	3,676,711	1.77235
SINE	42,379	0.020429	0	0	0	0	42,379	0.020429
LTR	7741029	3.731546	8,035,958	3.87372	33,888,591	1633592	37,751,938	16.752095
Other	1,901	0.000916	0	0	0	0	1901	0.000916
Unknown	0	0	0	0	14,408,869	6.945764	14,408,869	6.945764
Total	10,209,758	4.921591	8,663,620	4.17628	55,349,557	26681132	57,148,860	27.548482

Table S8. Summary of the annotated TEs in the *S. album* genome assembly

Class	Family	Length (bp)	Percent (%)
DNA	Academ	8878	0.0043
	CMC	2674158	1.2891
	CMC-Chapaev	9519	0.0046
	Crypton	107484	0.0518
	DNA	1938185	0.9343
	Dada	97167	0.0468
	Ginger	89908	0.0433
	Harbinger	164	0.0001
	Helitron	530216	0.2556
	IS3EU	35511	0.0171
	Kolobok	308009	0.1485
	MULE	1064495	0.5131
	Maverick	175624	0.0847
	Merlin	34390	0.0166
	MuLE	1217905	0.5871
	Novosib	5244	0.0025
	P	80958	0.0390
	PIF	251972	0.1215
	PiggyBac	9215	0.0044
	Sola	21095	0.0102
	TcMar	268317	0.1293
	Zator	96	0.0000
	Zisupton	6232	0.0030
	hAT	2151089	1.0369
LINE	Ambal	613	0.0003
	CR1	6950	0.0034
	DRE	2198	0.0011
	Dong-R4	769	0.0004
	I	1273	0.0006
	Jockey	8172	0.0039
	L1	2662270	1.2833
	L2	199706	0.0963
	LINE	4171	0.0020
	Penelope	50118	0.0242
	Proto1	1274	0.0006
	R1	102996	0.0496
	R2	452876	0.2183
	RTE	242443	0.1169
	Rex-Babar	1534	0.0007
	Tad1	505	0.0002
LTR	Cassandra	164587	0.0793
	Caulimoviru	358311	0.1727
	Caulimovirus	1621995	0.7819
	Copia	20667775	9.9629
	DIRS	14506	0.0070
	ERV	2125	0.0010
	ERV1	466639	0.2249
	ERV4	983	0.0005
	ERVK	40368	0.0195
	ERVL	2236	0.0011
	Gypsy	5461901	2.6329
	LTR	9098378	4.3859
	Ngaro	8039	0.0039
	Pao	15443	0.0074
Other	Composite	525	0.0003
	DNA_virus	1376	0.0007
SINE	5S	364	0.0002
	5S-Deu	168	0.0001
	5S-Sauria	624	0.0003
	B2	108	0.0001
	B4	2855	0.0014
	ID	2821	0.0014
	MIR	59	0.0000
	SINE	188	0.0001
	U	242	0.0001
	tRNA	34779	0.0168
	tRNA-Deu	443	0.0002
rRNA		585	0.0003
Satellite		460699	0.2221
Simple_repeat		188671	0.0909
Unknown		14408869	6.9458

Table S9. Statistics for TEs in several plant species

Species	Genome size (Mb)	TEs		Retroelements		DNA transposons		Other repeat sequences	
		Length (Mb)	% of genome	Length (Mb)	% of genome	Length (Mb)	% of genome	Length	% of genome
S. album	207.39	57.19	27.55	41.47	18.54	9.86	4.75	1901	0.31
M. oleifera	1510	1254.76	82.56	1053.96	69.35	64.79	4.26	34.73	2.3
V. vinifera	485.33	266.39	54.89	181.46	37.39	43.12	8.88	9.9	2.04
A. sinensis	783.8	467.57	64.35	257.13	35.39	41.21	5.67	28.84	3.68
O. sativa	373.25	185.63	49.73	98.55	26.40	61.91	16.59	21.05	5.64

Table S10. Statistics of predicted protein-coding genes in the *S. album* genome

**Method**		**Number**	**Average gene length (bp)**	**Average CDS length (bp)**	**Average exons per gene**	**Average exon length (bp)**	**Average intron length (bp)**
De novo	AUGSTUTUS	22,218	3,671.01	1,191.21	5.11	233.01	603.02
	GENSCAN	20,876	6,753.01	1,362.77	6.70	203.46	945.99
	SNAP	29,050	3,349.19	806.18	4.13	195.33	813.17
Homolog	*V. vinifera*	21,966	3,414.81	1,100.05	4.80	229.04	608.7
	A. thaliana	21,267	3,142.83	1,058.87	4.62	229.11	575.42
	P. trichocarpa	23,453	3,025.90	1,051.82	4.56	230.8	554.95
	O. sativa	20,100	2,966.04	1,034.76	4.31	240.13	583.61
RNA-seq	StringTie	24,465	24,465	2,362.37	6.21	380.4	634.95
Integration	EVM	23,283	3,812.22	1,188.93	5.40	220.36	596.82

Table S11. Summary of evidence for the EVM gene models for *S. album*

	≥ 20% overlap		≥ 50% overlap		≥ 80% overlap	
	**Number**	**%**	**Number**	**%**	**Number**	**%**
**P (single)**	135	0.5798	524	2.2506	2,061	8.8520
**P (more)**	2,676	11.4934	2,874	12.3438	2,714	11.6566
**H (single)**	4	0.0172	7	0.0301	25	0.1074
**H (more)**	4	0.0172	4	0.0172	70	0.3006
**C (single)**	5	0.0215	7	0.0301	179	0.7688
**H + C**	3	0.0129	28	0.1203	589	2.5297
**P + C**	1,207	5.1840	1,658	7.1211	2,864	12.3008
**P + H**	4,804	20.6331	4,513	19.3832	3,808	16.3553
**P + H + C**	14,445	62.0410	13,338	57.2864	10,877	46.7165

P: *ab initio* prediction; H: homology-based; C: RNA-seq expressed genes. According to the number of gene models supported (P, H or C), the evidence was further separated into single genes (with one gene source) and more (with two or more gene sources). The overlap threshold is relative to the CDS region of EVM genes.

Table S12. Functional annotation of the predicted genes

**Database**	**Number**	**Percent (%)**
Nr	20,485	87.98
Swiss-Prot	16,856	72.40
TrEMBL	20,447	87.82
InterPro	20,249	86.97
KEGG	16,160	69.41
COG	8,456	36.32
GO	12,354	53.06
Annotated	20,880	89.68
Total	23,283	100

Table S13. Statistics for non-coding RNA genes in the *S. album* genome

Class	Type	Copy (w*)	Average length (bp)	Total length (bp)	% of genome
miRNA		65	129.0923	8,391	0.0040
tRNA		495	75.3131	37,280	0.0180
rRNA	18S	85	798.2471	67,851	0.0327
	28S	144	138.4861	19,942	0.0096
	5.8S	40	147.1000	5,884	0.0028
	5S	316	112.1709	35,446	0.0171
	total	585	220.7231	129,123	0.0622
snRNA	CD-box	127	93.4409	11,867	0.0057
	HACA-box	38	129.5789	4,924	0.0024
	Splicing	92	155.9239	14,345	0.0069
	total	257	121.1518	31,136	0.0150

Note: w* whole genome annotation, comprehensive data to calculate the average length and total length.

Table S14. Protein datasets used for gene family analysis

Species	Reference
Malania oleifera	http://dx.doi.org/10.5524/100549
Arabidopsis thaliana	https://www.arabidopsis.org/
Oryza sativa	http://rice.plantbiology.msu.edu/
Populus trichocarpa	http://plants.ensembl.org/Populus_trichocarpa/Info/Index
Solanum lycopersicum	https://solgenomics.net
Vitis vinifera	https://plants.ensembl.org/index.html (V51)
Aquilaria sinensis	http://gigadb.org/dataset/100702
Lonicera japonica	https://ngdc.cncb.ac.cn/search/?dbId=gwh&q=GWHAAZE00000000
Antirrhinum majus	http://bioinfo.sibs.ac.cn/Am/index.php
Myrica rubra	https://ftp.ncbi.nlm.nih.gov/genomes/all/GCA/003/952/965/GCA_003952965.2_Mru_ZJU_2/
Cucumis sativus	http://cucurbitgenomics.org/ftp/genome/cucumber/Chinese_long/v3/
Citrus sinensis	https://www.citrusgenomedb.org/
Malus domestica	http://dx.doi.org/10.5524/100189

Table S15. Corresponding relation of Chromosome IDs documented by this study and previous report

	Chromosome ID	
	**In this study***	**Hong et al. (2023)**
1	SaChr01	SALChr10
2	SaChr02	SALChr03
3	SaChr03	SALChr04
4	SaChr04	SALChr02
5	SaChr05	SALChr01
6	SaChr06	SALChr07
7	SaChr07	SALChr08
8	SaChr08	SALChr09
9	SaChr09	SALChr05
10	SaChr10	SALChr06

*All genes were named according to the nomenclature used for Arabidopsis (Arabidopsis Genome Initiative, 2000) to indicate the relative positions of genes on the pseudochromosomes.

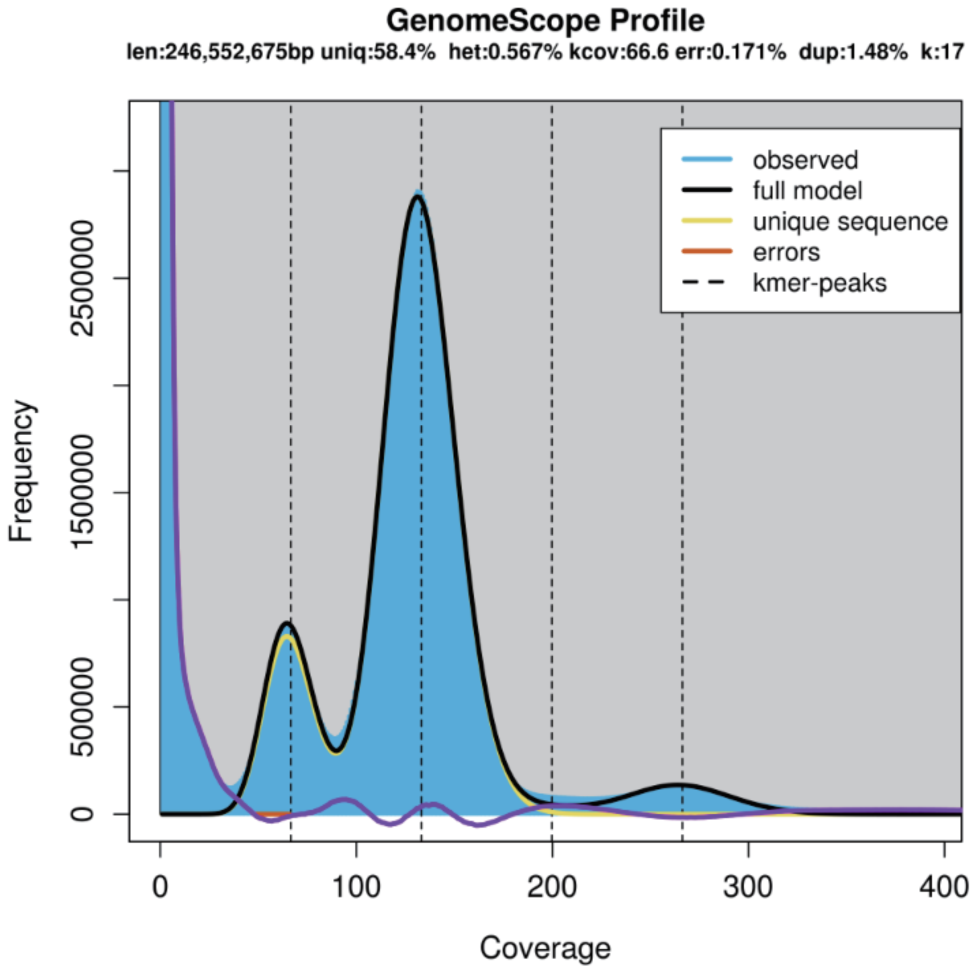

Figure S1. Size estimation of the *S. album* genome. Genome size was estimated by calculating the distribution of 17-mer frequency in the sequencing reads. The x-axis is the depth (X), and the y-axis is the proportion of sequences that represent the frequency at that depth divided by the total frequency of all depths.

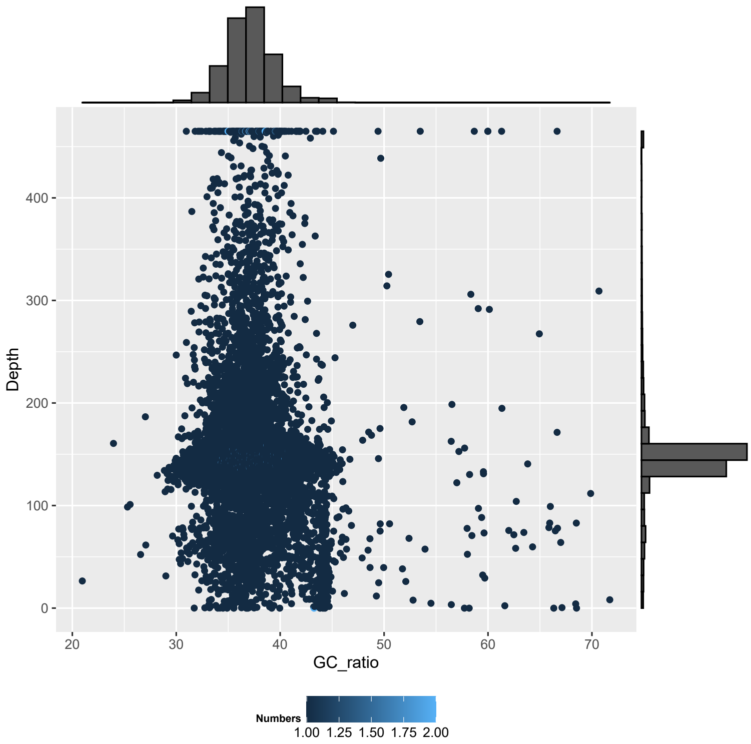

Figure S2. Relationship between percent of GC content and sequencing depth.

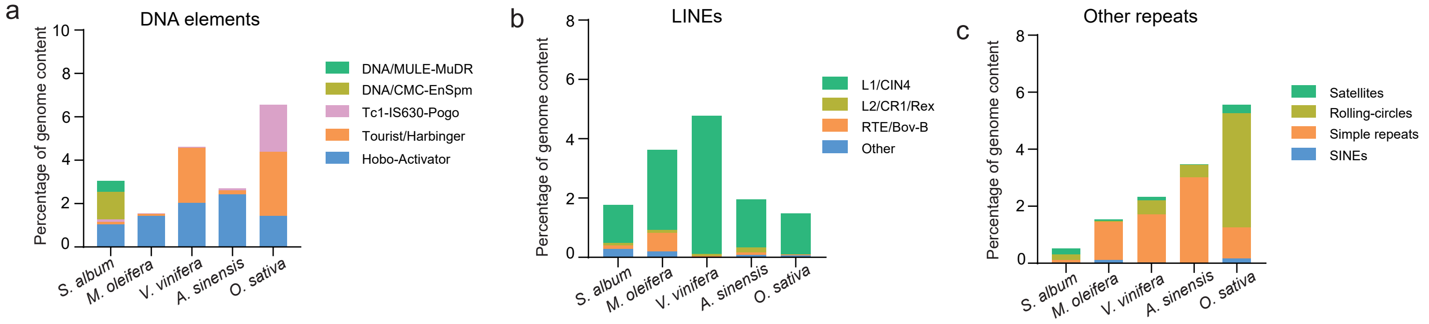

Figure S3. Distribution of repeat elements across five plant species. Percentage of genome content consisting of DNA element repeats (a), LINEs repeat (b), and other repeats (c).

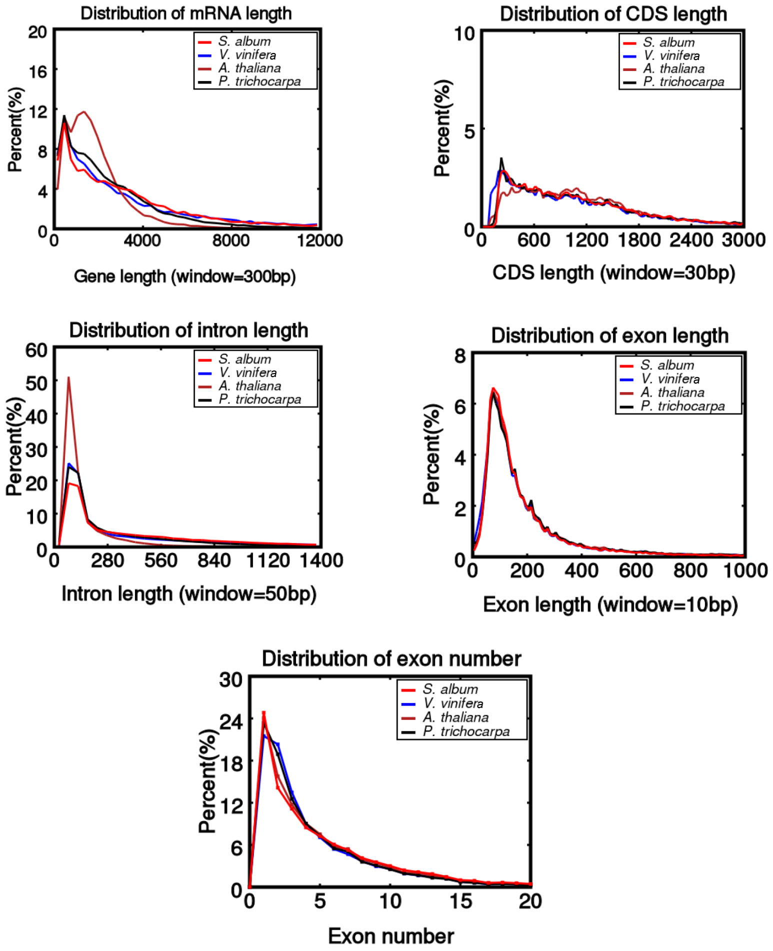

Figure S4. Comparisons of gene features between *S. album* tree and three other plant species.

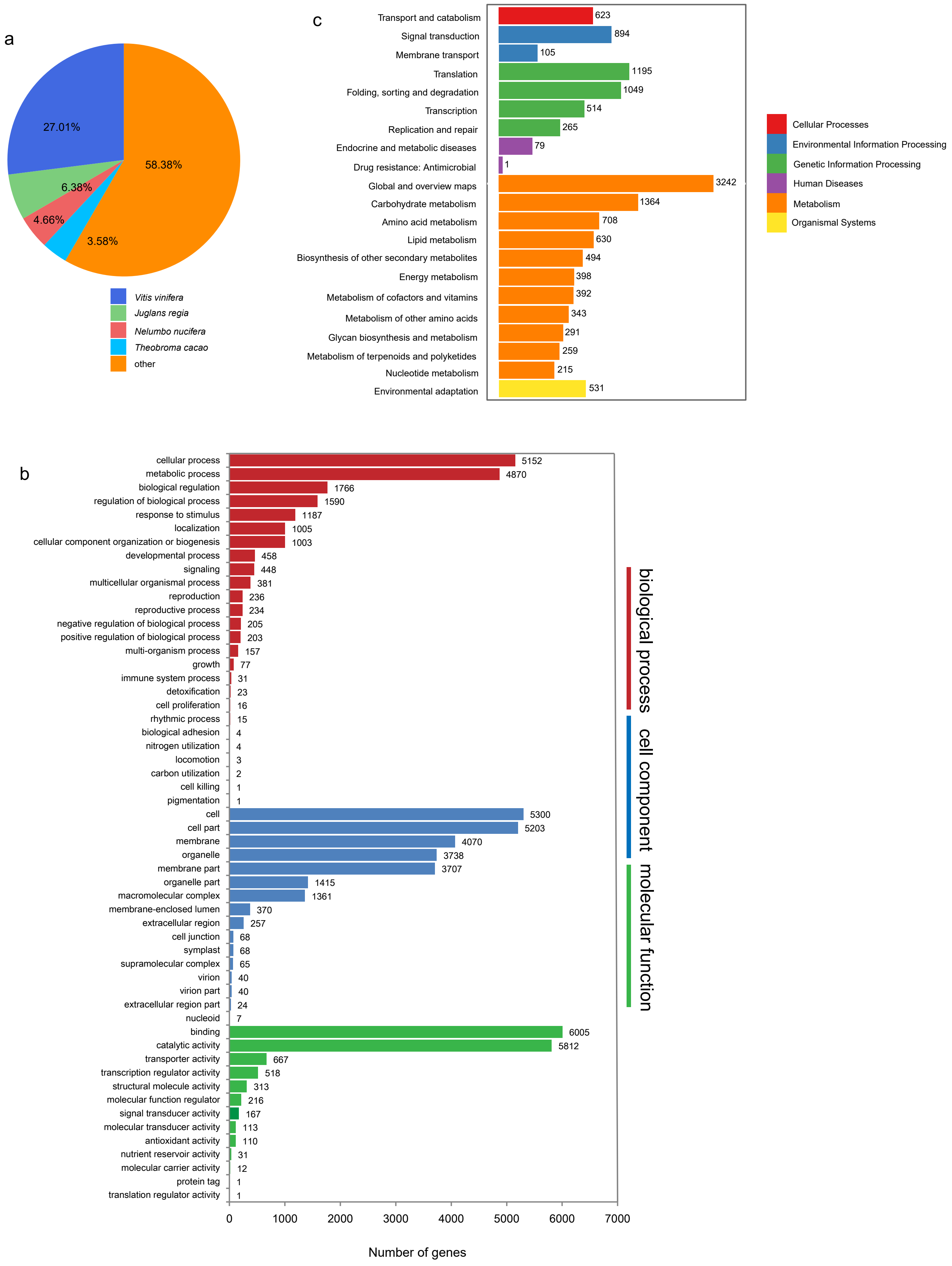

Figure S5. Annotation of the sandalwood genome. (a) Nr homologous species distribution of the predicted genes. (b) GO functional enrichment of sandalwood genes. (c) KEGG pathway enrichment distribution of the sandalwood genes.

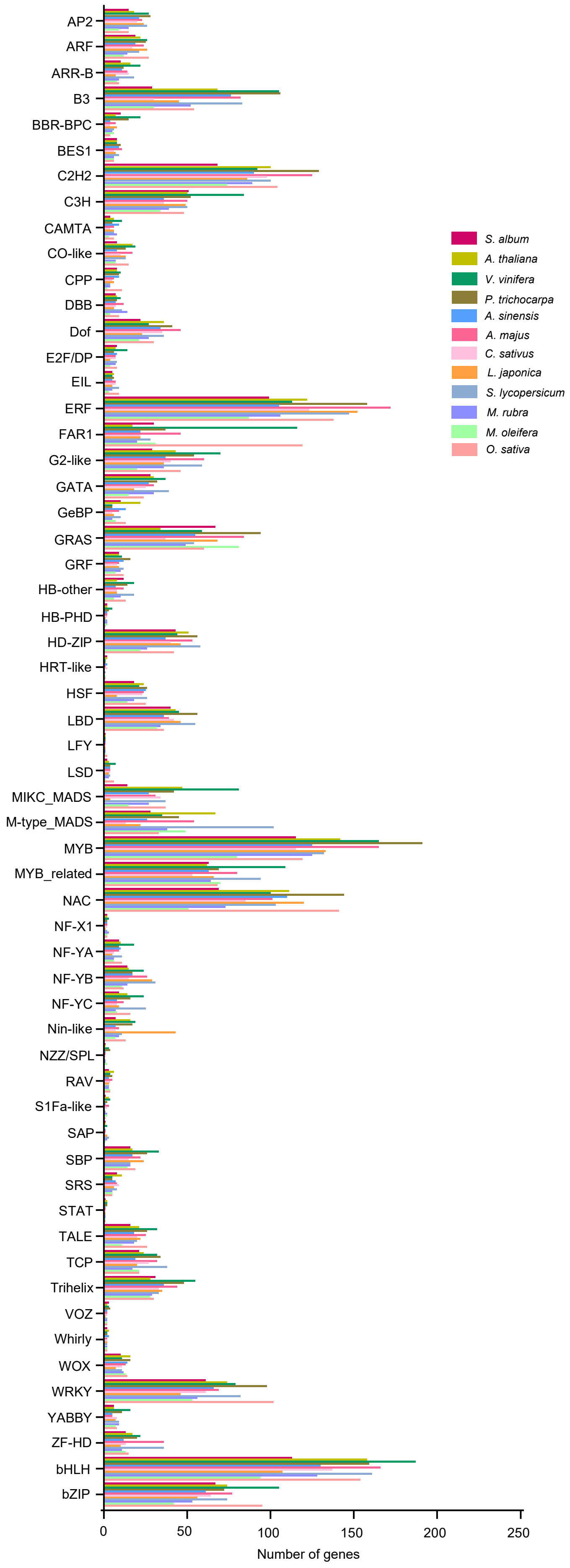

Figure S6. Comparison of classification of transcription factors in *S. album* and 11 selected plants.
